# Preclinical Drug Pharmacokinetic, Tissue Distribution and Excretion Profiles of the Novel Limonin Derivate HY-071085 as an Anti-Inflammatory and Analgesic Candidate in Rats and Beagle Dogs

**DOI:** 10.3390/ph15070801

**Published:** 2022-06-27

**Authors:** Liping Dong, Wenjuan Liu, Xiaoyuan Zhao, Feng Yu, Yungen Xu, Mengxiang Su

**Affiliations:** 1 School of Basic Medicine and Clinical Pharmacy, China Pharmaceutical University, Nanjing 211198, China; 13512538014@163.com; 2 School of Pharmacy, China Pharmaceutical University, Nanjing 211198, China; 3321010047@stu.cpu.edu.cn (W.L.); 3320011021@stu.cpu.edu.cn (X.Z.)

**Keywords:** limonin, HY-071085, pharmacokinetics, oral bioavailability, tissue distribution, excretion

## Abstract

Limonin is one of the research hotspots in natural drug development. However, its low solubility in water leads to poor oral bioavailability, discouraging the further study of its potential as a candidate compound. In order to overcome this limitation, and to enhance its biological activities, a novel limonin derivative—HY-071085—was synthesized by structural modification, and has exhibited strong anti-inflammatory and analgesic activity. In order to achieve a thorough understanding of the biological actions of HY-071085 in vivo, this study evaluated the pharmacokinetics and bioavailability of HY-071085 in rats and beagle dogs, and the distribution and excretion in rats. Using ultra-high-performance liquid chromatography-tandem mass spectrometry, the kinetic profiles of HY-071085 in the plasma of healthy rats and beagle dogs after a single gavage, repeated gavages and the intravenous injection of HY-071085 were studied. The tissue distribution (heart, liver, spleen, lung, kidney, gastric tissue, intestine, brain, skin, testis, ovary and womb) and excretion of HY-071085 were also studied. These results showed that HY-071085 has nonlinear dynamic characteristics in rat and beagle dog plasma. It was found that the plasma concentrations of HY-071085 in female rats were significantly higher than those in male rats after a single oral administration. There were gender differences in the kinetic behavior of HY-071085 in rats; however, there was no difference identified in dogs. HY-071085 was mainly eliminated as metabolites in rats, and was distributed in most of the tissues except the brain, with the highest content being in the gastric tissue and intestinal arease, followed by the liver, spleen, fat, lung, kidney, ovary and heart. The bioavailability of HY-071085 in male and female rats was 2.8% and 10.8%, respectively, and was about 13.1% in beagle dogs. The plasma protein binding rate of HY-071085 in rats, beagle dogs and humans ranged from 32.9% to 100%, with obvious species differences. In conclusion, our study provides useful information regarding the absorption, distribution and excretion of HY-071085, which will provide a good base for the study of the mechanism of its biological effects.

## 1. Introduction

**Figure 1:**
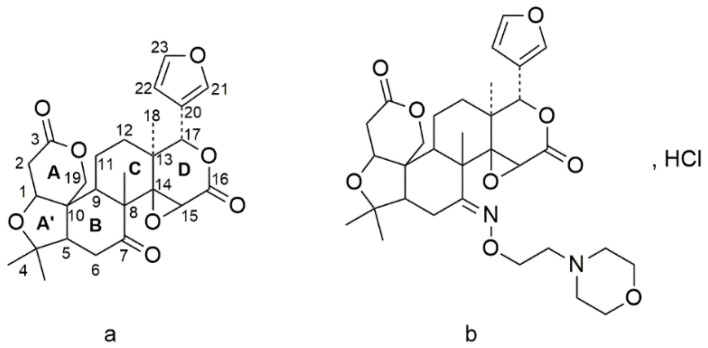
The chemical structures of limonin (**a**) and HY-071085 (**b**).

Limonoids are highly oxygenated triterpenoids, and over 300 limonin analogs have been isolated from natural sources with various therapeutic effects [1,2]. Limonin (Figure 1a) is representative of those compounds, and is the most abundant limonoid in the natural environment. Since limonin was first identified by Matsuda [3,4], various studies have been performed to explore its possible pharmacological activities [5], such as anti-inflammatory and analgesic [4,6,7,8,9,10], anticancer [11,12], antioxidant [13], antimicrobial [14], antiviral [15], liver protection [16] and antiallergy effects [17], etc. However, the low solubility in water of limonin leads to poor oral bioavailability, discouraging further study of it as a candidate compound [18]. In order to improve its efficacy and water-solubility, the creation of novel limonin derivatives by structural modification has become one of the key focus areas. In our previous studies, analogues were obtained by introducing various tertiary amines onto the C (7)-position [10,19] A ring [20,21] of limonin or deoxylimonin, which possessed favorable physicochemical and water-soluble properties. Furthermore, some of them exhibited analgesic activities that were more potent than those of aspirin, and anti-inflammatory activities that were stronger than those of naproxen. Those results have encouraged the further development of some of them as potential candidates for the treatment of inflammatory diseases [6]. HY-071085 (Figure 1b), a novel water-soluble derivative of limonin, (12S,12aS,E)-12-(furan-3-yl)-6,6,8a,12a-tetramethyl-8-((2-morpholinoethoxy)imino)dodecahydro-1H,3H-oxireno [2,3-d]pyrano [4’,3’:3,3a]isobenzofuro [5,4-f]isochromene-3,10(9aH)-dione, has demonstrated strong anti-inflammatory and analgesic activity in both in vitro and in vivo studies [10].

Previously, several pharmacokinetic studies of limonin in rats [22,23], beagle dogs [24] and humans [18,25] have showed that oral absorption was very poor, and only a small amount of the drug entered the blood, which may have mainly been due to its insoluble chemical structure. Moreover, limonin was gradually metabolized after entering the blood, and the elimination half-life was short [22,24,26]. Currently, research on the structure–activity relationship and pharmacological effects of limonin analogues is very in-depth [1,5], but there are few studies on their pharmacokinetics, and it is not clear whether the structural modifications ameliorate their pharmacokinetic profiles. Pharmacokinetic studies of HY-071085 in experimental animals are essential for the further development of HY-071085. The preclinical pharmacokinetic characteristics of HY-071085 are necessary for a comprehensive understanding of its efficacy and potential toxicity. In the current study, we used a rapid, selective and highly sensitive LC–MS/MS method with simple pretreatment procedures to quantify HY-071085 in rat and beagle dog plasma, urine, and tissue in only 3.5 min with an LLOQ of 1.0 ng/mL. The preclinical pharmacokinetics, tissue distribution and excretion of HY-071085 in urine, feces and bile were investigated by this method.

## 2. Results

### 2.1. Pharmacokinetics after a Single Dose of HY-071085 in Rats

The area under the curve (AUC), mean residence time (MRT), elimination half-life(t_1/2_), clearance (Cl/F) and steady-state apparent distribution volume (Vss/F) were calculated by the method of moments after suspensions of low (3.25 mg/kg), medium (6.5 mg/kg) and high (13 mg/kg) amounts of HY-071085 (before use, ground with 0.5% sodium carboxymethylcellulose solution) were administered by gastric gavage to male and female rats. Blood samples were collected from the fundus venous plexus at various times within 8 h after administration in order to analyze the concentration of HY-071085 in plasma.

**Figure 2:**
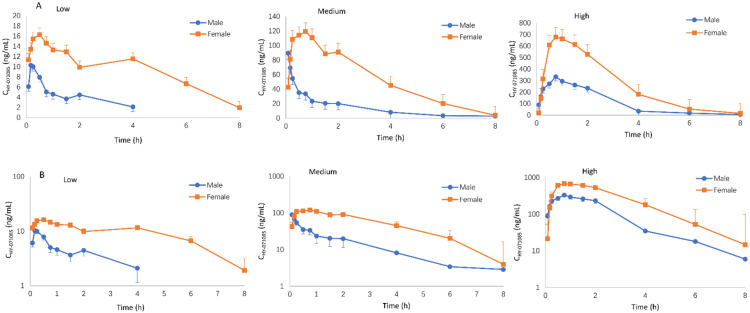
Mean plasma concentration time curve of HY-071085 after the single-dose intragastric administration of low (3.25 mg/kg), medium (6.5 mg/kg) and high (13 mg/kg) doses of HY-071085 in rats. (**A**) Constant coordinates; (**B**) semilogarithmic coordinates.

**Figure 3:**
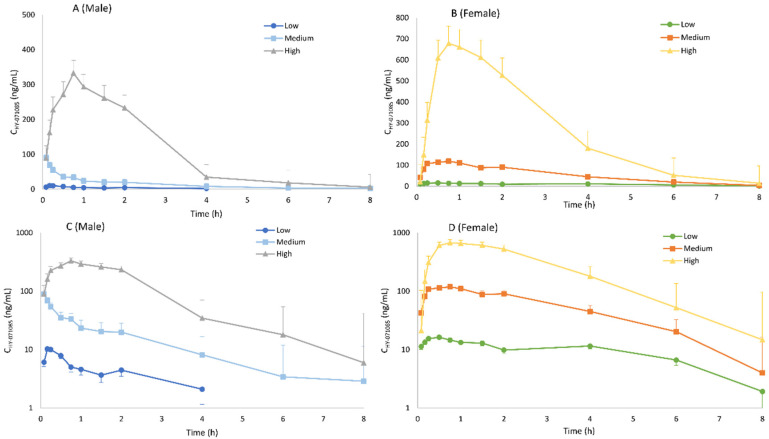
Comparison of the mean plasma concentration–time curve of HY-071085 after the single-dose intragastric administration of low (3.25 mg/kg), medium (6.5 mg/kg) and high (13 mg/kg) doses of HY-071085 in rats. (**A**,**B**) Constant coordinates; (**C**,**D**) semilogarithmic coordinates.

The profiles of the mean plasma concentration of HY-071085 versus time after a single dose in rats are presented in Figure 2 and Figure 3, and the PK parameters are listed in Table 1. The relationships between C_max_, AUC and the dose are shown in Figures S1 and S2, respectively. The exposures of male rats in the low-, medium- and high-dose groups of HY-071085 was significantly lower than those of female rats. At the three doses, the AUC_tn_ ratios of the female rats and male rats were 3.5, 3.9 and 2.4, respectively. The AUC_tn_ ratio among male rats was 1:5.16:41.06, and among female rats was 1:5.71:28.20. The results showed that the increased proportion of the AUC was greater than the dose, and that the clearance rate decreased significantly, indicating that the drug showed obvious nonlinear metabolic kinetics within the investigated dose range.

**Figure 4:**
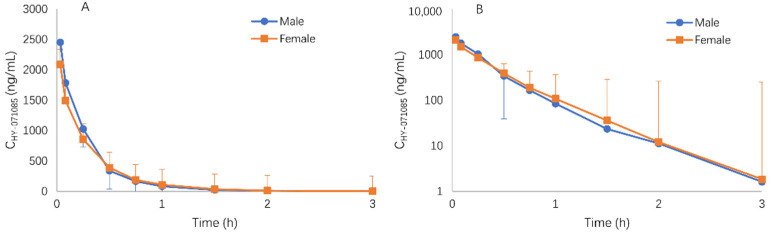
Mean plasma concentration–time curve of HY-071085 after the single-dose intravenous administration of 3.25 mg/kg HY-071085 in rats. (**A**) Constant coordinates; (**B**) semilogarithmic coordinates.

The PK parameters after the intravenous administration of 3.25 mg/kg HY-071085 (before use, prepared with 5% DMSO + 95% normal saline mixture) are listed in Table 1. In contrast to intragastric administration, there were no gender differences in the plasma concentration time data (Figure 4) and PK parameters, indicating that the gender differences from intragastric administration mainly result from the process of absorption. According to the AUCs of 3.25 mg/kg HY-071085 by intragastric and intravenous administration, the calculated absolute oral bioavailability of HY-071085 in male and female rats were 2.8% and 10.8%, respectively.

### 2.2. Pharmacokinetics after Repeated Doses of HY-071085 in Rats

**Figure 5:**
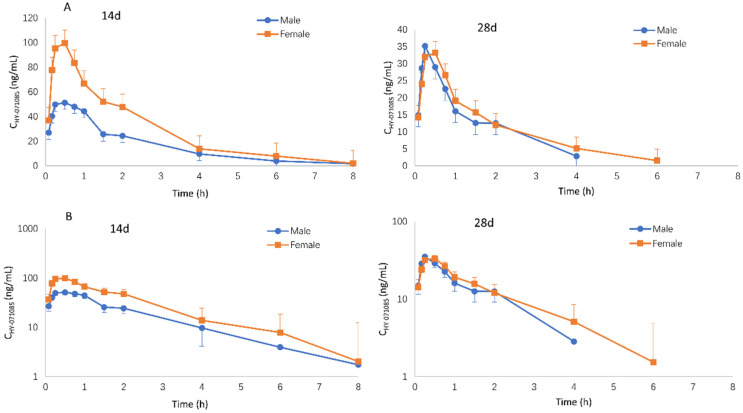
Mean plasma concentration–time curves of HY-071085 after repeated intragastric doses of 6.5 mg/kg in rats. (**A**) Constant coordinates; (**B**) semilogarithmic coordinates.

**Figure 6:**
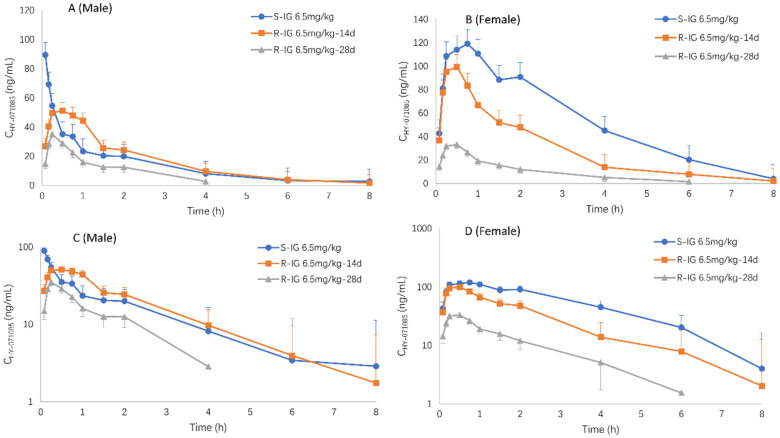
Comparison of the mean plasma concentration–time curves of HY-071085 after single and repeated intragastric doses of 6.5 mg/kg HY-071085 in rats. (**A**,**B**) Constant coordinates; (**C**,**D**) semilogarithmic coordinates.

Male and female rats were given HY-071085 (6.5 mg/kg) by gastric gavage twice a day for 28 days. On days 14 and 18, blood samples were collected from the fundus venous plexus at various time points before and within 8 h after administration in order to analyze the concentration of HY-071085 in the plasma (Figure 5). The PK parameters are listed in Table 1. The results showed that the plasma drug exposure level of female rats had a downward trend compared with that of a single dose after 14 days of continuous administrations of 6.5 mg/kg per day; however, there was no significant change in the male rats. After 28 days of continuous administration, the plasma exposure levels of the male and female rats decreased by 49% and 85%, respectively, compared with that after a single administration (Figure 6). The results suggested that multidose administration may induce metabolic enzymes, leading to a significant decrease in the exposure level.

### 2.3. Pharmacokinetics after Single Doses of HY-071085 in Dogs

**Figure 7:**
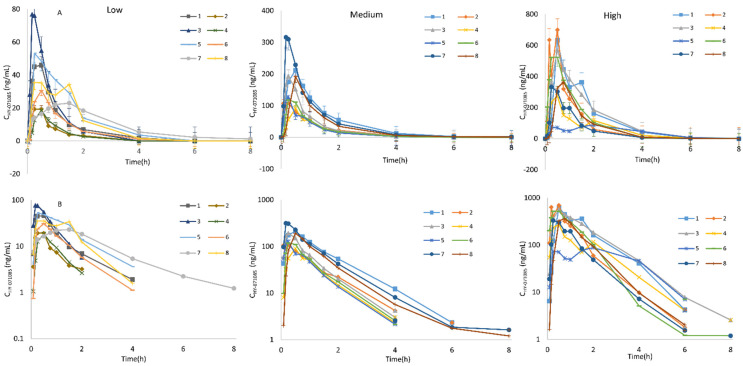
Mean plasma concentration–time curve of HY-071085 after the single-dose intragastric administration of HY-071085 at low (1.25 mg/kg), medium (2.5 mg/kg) and high (5 mg/kg) doses in beagle dogs. (**A**) Constant coordinates; (**B**) semilogarithmic coordinates.

**Figure 8:**
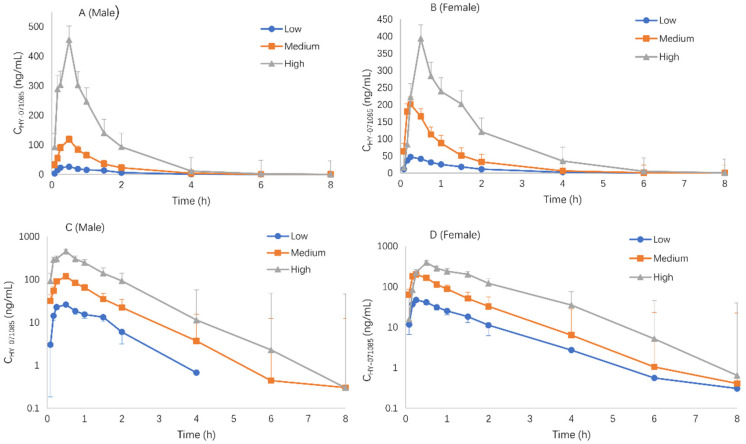
Comparison of the mean plasma concentration–time curves of HY-071085 after the single-dose intragastric administration of HY-071085 at low (1.25 mg/kg), medium (2.5 mg/kg) and high (5 mg/kg) doses in beagle dogs. (**A**,**B**): Constant coordinates; (**C**,**D**): semilogarithmic coordinates.

Male and female beagle dogs were administered low (1.25 mg/kg), medium (2.5 mg/kg) and high (5 mg/kg) doses of HY-071085 by gastric gavage, and blood samples were collected from the forelimb vein at various time points before and 8 h after administration in order to analyze the concentration of HY-071085 in the plasma. The profiles of the mean plasma concentration of HY-071085 versus time in dogs are presented in Figure 7, and the PK parameters are listed in Table 2. There was no gender difference in the pharmacokinetics in dogs (Figure 8). The AUC_tn_ ratio for the three doses was 1:3.8:11.9, and the AUC_0-∞_ ratio between doses was 1:3.6:11.3, which was greater than the dose increase ratio (1:2:4), as shown in Figures S3 and S4. The results showed that the clearance rate decreased significantly, suggesting that HY-071085 has obvious nonlinear metabolic kinetics within the investigated dose range.

**Figure 9:**
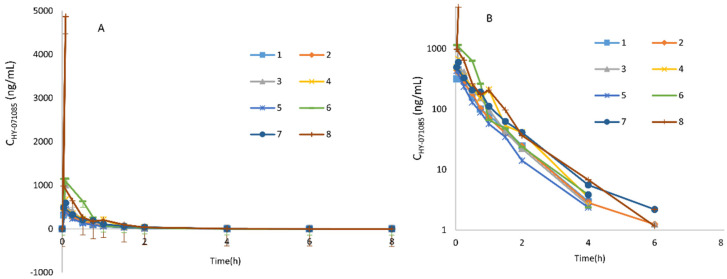
Mean plasma concentration–time curve of HY-071085 after the single-dose intravenous administration of 1.25 mg/kg HY-071085 in beagle dogs. (**A**) Constant coordinates; (**B**) semilogarithmic coordinates.

After the intravenous administration of 1.25 mg/kg HY-071085, there was also no gender difference for the plasma concentration time data in Figure 9. According to the AUC_0-∞_ of HY-071085 after 1.25 mg/kg intragastric and intravenous administration, the calculated absolute oral bioavailability of HY-071085 was 13.1% in beagle dogs, which was similar to that in female rats.

### 2.4. Pharmacokinetics after Repeated Doses of HY-071085 in Dogs

**Figure 10:**
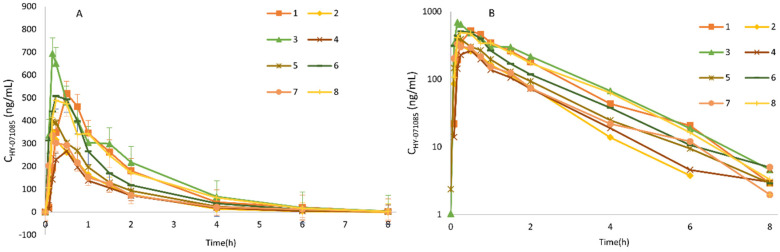
Plasma concentration–time curve of HY-071085 after the continuous repeated-dose intragastric of 2.5 mg/kg in beagle dogs. (**A**) Constant coordinates; (**B**) semilogarithmic coordinates.

**Figure 11:**
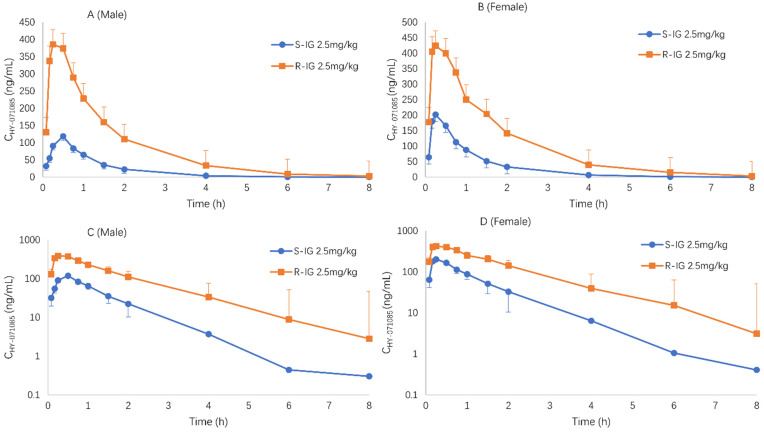
Comparison of the mean plasma concentration–time curves of HY-071085 after single and repeated intragastric doses of 2.5 mg/kg in beagle dogs. (**A**,**B**) Constant coordinates; (**C**,**D**) semilogarithmic coordinates (*n* = 8).

The t_1/2_ of HY-071085 after the single- and repeated-dose intragastric administration of 2.5 mg/kg in beagle dogs was 0.83 ± 0.12 and 1.08 ± 0.13 h, respectively. AUC_0-∞_ was 195.41 ± 90.03 and 728.57 ± 270.64 h∙ng/mL, respectively (Table 2, Figure 10). Compared with the single dose, the plasma exposure level of HY-071085 increased significantly after repeated-dose administration (Figure 11). On the fifth and seventh days before administration, the plasma concentration (valley concentration) was mostly below the lower limit of quantification (1 ng/mL).

### 2.5. Tissue Distribution in Rats

**Figure 12:**
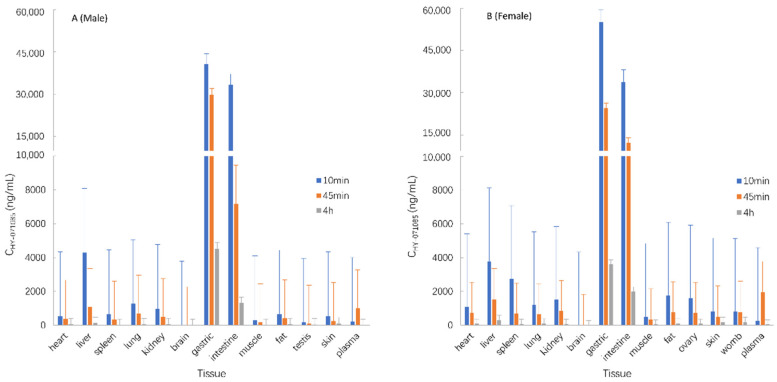
The drug concentration in tissues (ng/mL) after the intragastric administration of 13 mg/kg HY-071085 for 10 min, 45 min and 4 h ((**A**) male; (**B**) female).

The male and female rats were euthanized by femoral artery bleeding under anesthesia after the single-dose intragastric administration of HY-071085 (13 mg/kg) for 10 min, 45 min and 4 h, and the tissues were separated for the analysis of the concentration of HY-071085 in the homogenate. The results showed that HY-071085 was mainly distributed in the gastric, intestine, liver, spleen, lung and kidney tissues, and less so in the ovary, womb, testis and other tissues (Figure 12).

### 2.6. Excretion in Rats

After the single-dose intragastric administration of HY-071085 (6.5 mg/kg) to male and female rats, their feces, urine and bile were collected at various time points within 24 h in order to analyze the excretion of HY-071085. The amount of unchanged parent compound recovered from the feces, urine and bile was negligible in rats (about 1.3% of the dose, Table 3).

### 2.7. Plasma Protein Binding

The evaluation of 50 ng/mL, 100 ng/mL, 200 ng/mL and 400 ng/mL doses of HY-071085 in human, dog and rat plasma showed that there were obvious species differences in the plasma protein binding (PPB) rates of HY-071085, which ranged from 32.9% to 100%. The PPB rate in dogs was about 40% at different concentrations in the concentration range (50–400 ng/mL), and in rats it was about 75%. The PPB rate in human plasma varied greatly at different concentrations, ranging from 100% at a low drug concentration to 58% at a high drug concentration, as shown in Table 4.

## 3. Discussion

After the single-dose intragastric administration of HY-071085 in rats, there were gender differences in the kinetic behavior of HY-071085. The drug exposure of female rats was about three times that of male rats. Similarly, the PK profile of limonin in rats demonstrated the existence of marked gender differences. The exposures of the female rats were 50-fold higher than those in the male rats after oral administration, and three-fold higher after intravenous administration [27]. Furthermore, the AUC was not directly proportional to the dosage, and HY-071085 showed significant nonlinear kinetic characteristics in rats. The results in beagle dogs after the single-dose intragastric administration of HY-071085 were consistent with those of rats, and also showed nonlinear dynamic characteristics. However, unlike in rats, there were no gender differences in the PK behavior in dogs. During 28 days of intragastric administration, the plasma drug exposure level of female rats decreased on the 14th day and decreased significantly on the 28th day (about 15% of the single dose). There was no significant change in the male rats on the 14th day; however, the exposure level decreased significantly on the 28th day (about 50% of the single dose). In contrast to the rats, the plasma exposure level of beagle dogs increased significantly after the repeated-dose intragastric administration of HY-071085. However, compared with limonin, the half-life of HY-071085 in rat and beagle dog plasma was significantly shorter [22,24,26,28,29].

The results showed that repeated-dose administration may induce metabolic enzymes, and the induction increased with the increase of the administration time, leading to a significant decrease in the plasma exposure level. The changes in plasma drug exposure may also be related to the speed of metabolism or absorption. In addition, the differential expression of hepatic drug-metabolizing enzymes may be a nonnegligible reason for the gender difference. The differences in metabolic elimination of liver microsomes in different species’ in vitro metabolic capacity of HY-071085 may provide an explanation for the species differences in the pharmacokinetics [30,31].

The bioavailability of HY-071085 in male and female rats was 2.83% and 10.8%, respectively. This suggests that the bioavailability of the drug was not high, and there were significant gender differences in rats. The bioavailability of HY-071085 in beagle dogs was about 13.1%, which was similar to that in female rats (10.8%). It seemed that that the structural modifications did not ameliorate the bioavailability of limonoids. It is necessary to consider the improvement of the bioavailability in subsequent formulation designs. However, compared with limonin, the time for HY-071085 to reach the maximum plasma concentration in rat and beagle dog plasma was significantly shortened [24,28,29,32]. This may be related to the increase of the distribution coefficient (log*D*) and partition coefficient (log*P*) values of HY-071085 (Table S6) [10].

The concentration of HY-071085 in the brain was extremely low, indicating that the drug could not easily penetrate the blood–brain barrier. The study on the excretion and material balance of HY-071085 in rats found that the drug was mainly eliminated by metabolism in rats. The plasma protein binding rate of HY-071085 had obvious species differences, and the plasma protein binding rate was between 32.9% and 100% in rats, dogs and humans. The plasma protein binding rate in dogs was lower than that in rats. There was a nonlinear binding phenomenon in the human plasma protein binding rate, and with the increase of the concentration, the protein binding rate decreased from 100% to about 58%. These cross-species differences introduced uncertainty in the human PK and dose projections.

## 4. Materials and Methods

### 4.1. Chemicals and Reagents

The HY-071085 reference substance (lot 20190316, 99.9% HPLC pure) and HY-071085 active pharmaceutical ingredient (lot TM-JP-20190424) were prepared by Hefei Medical Industry Pharmaceutical Co., Ltd. (Hefei, China). The internal standard (IS), theophylline standard (lot 100121–201805), was purchased from the National Institute for the Control of Pharmaceutical and Biological Products (Beijing, China). LC-MS grade methanol and acetonitrile were purchased from Merck Company Inc. (Rahway, NJ, USA). Human plasma was provided by Jiangsu Provincial Blood Center (Nanjing, China). Deionized water was prepared through a Milli-Q Gradient A10 ultrapure water device (Millipore, Burlington, MA, USA). The other reagents were commercially available and analytically pure.

### 4.2. Instrumental Conditions for UPLC-MS/MS

The UPLC-MS/MS system consisted of the ExionLC Series and SCIEX Triple Quad 4500 equipped with an electrospray ionization (ESI) source (AB, Framingham, MA, USA). The chromatographic separation was performed using a Shim-pack VP-ODS column (250 mm × 2.0 mm I.D, 4.6 μm, Shimadzu, Japan). The isocratic mobile phase was 0.01% formic acid in water–acetonitrile (72:28, *v*/*v*) at a flow rate of 0.2 mL/min. In order to minimize the contamination of the ESI source and the mass spectrometer, the eluent was switched to a waste line during the first minute. The total run time was 3.5 min. The auto-sampler temperature was adjusted to 4 °C to achieve optimal stability.

Multiple reaction monitoring (MRM) was performed using an ESI source in positive ion mode. The instrumental conditions were as follows: curtain gas (35 psi), collision gas (8 psi), ionspray voltage (5500 V), temperature (550 °C), ion source gas 1 (55 V), ion source gas 2 (55 V), entrance potential (10 psi) and collision cell exit potential (6 psi). The declustering potentials for HY-071085 and theophylline (IS) were 150 V and 84 V, respectively. The collision energies for HY-071085 and theophylline were 75 V and 27 V, respectively. The precursor ([M+H]^+^)-to-product ion transitions used for quantification were *m/z* 599.3→114.1 for HY-071085 and *m/z* 181.1→124.0 for theophylline.

### 4.3. Animals and Study Design

The animal studies were approved by the Animal Experiment Ethics Committee of the China Pharmaceutical University, and all of the studies were conducted according to approved animal care protocols. All of the animals were separately housed in stainless steel cages in a ventilated animal room with a temperature of 20–24 °C (rats) or 20–29 °C (dogs) and a relative humidity of 40–70%, with lighting 12 h/day. Water was freely available for all of the animals.

In vivo PK profiling in the rats: Sprague Dawley rats (approximately 177–221 g in weight) were purchased from Shanghai Xipur-Bikai Experimental Animal Co., Ltd. (Shanghai, China). Before the experiment, several rats were randomly divided into several groups, half males and half females in each group, fasting but free to drink water for 12 h. The pharmacokinetic study design is summarized in Table 5. The dosage of the rats was dependent on the drug pharmacology: the HY-071085 active pharmaceutical ingredients were administered with a dosage volume of 0.25 mL/100 g body weight. The whole-blood samples were centrifuged at 8000 rpm for 5 min, and the plasma samples were separated and stored at –80 °C until the analysis.

In vivo PK profiling in dogs: Beagle dogs (approximately 8–10 kg in weight) were purchased from Jiangsu Yadong Experimental Animal Research Institute Co. Ltd (Nanjing, China). Before the experiment, several beagle dogs were randomly divided into several groups, half males and half females in each group. Four cycles of single-dose administration experiments were carried out, and the cleaning period was 3–5 days. Repeated-dose administration was carried out after the end of four cycles of single-dose administrations and cleaning periods. The pharmacokinetic study design is summarized in Table 6. According to the dosage for the rats, the dosage for the beagle dogs was converted via the body surface area method. The HY-071085 active pharmaceutical ingredients were administered with a dosage volume of 2 mL/kg body weight. The whole-blood samples were centrifuged at 8000 rpm for 5 min, and the plasma samples were separated and stored at –80 °C until the analysis.

Tissue distribution in rats: Rats were randomly allocated into three groups (10 rats/group) for the tissue distribution studies. These rats were sacrificed at 10 min, 45 min and 4 h after orally administering HY-071085. Fourteen organs—the heart, liver, spleen, lung, kidney, stomach, gut, muscle, fat, skin, brain, ovary (female), womb (female) and testis (male)—were removed, washed in normal saline, and dried with filter papers immediately. The organs were accurately weighed and individually homogenized with an appropriate amount of deionized water. The tissue homogenate was stored at –80 °C until analysis.

Excretion in urine and feces (rats)*:* The Sprague Dawley rats were aged approximately 6 weeks old and weighed 185–212 g at the onset of treatment. Urine and fecal samples were collected for the excretion study using metabolic cages. After the oral administration of 6.5 mg/kg HY-071085, urine and feces were collected at 0–6, 6–12 and 12–24 h. The urine volumes were recorded and transferred to centrifuge tubes. The fecal weights were recorded at each sampling period, and then those feces were dried, ground and mixed thoroughly. Then, 0.1 g feces was accurately weighed, mixed with 1 mL deionized water, and homogenized to be used as a fecal sample. All of the samples were stored at –80 °C until the analysis.

Excretion in bile (rats): The Sprague Dawley rats were approximately 6 weeks old and weighed 174–215 g at the onset of treatment. The rats were anesthetized with diethyl ether, and the bile duct was then cannulated for bile collection. After allowing recovery from surgery and anesthesia, these bile duct–cannulated rats were administered a single 6.5 mg/kg dose of HY-071085 by gastric gavage.

### 4.4. Preparation of the Calibration Standards and QC Samples

Standard stock solutions of HY-071085 and theophylline were prepared by dissolving each drug in acetonitrile with nominal concentrations of 1.06 mg/mL and 1.01 mg/mL, respectively. The standard working solution of HY-071085 was prepared by appropriately diluting the stock solution with acetonitrile, and the concentrations were 0.005, 0.01, 0.025, 0.1, 0.25, 1, 2.5, 4, 5 and 100 μg/mL, respectively. The theophylline working solution was prepared at a concentration of 100 ng/mL from stock solution using acetonitrile for dilution. The final concentrations of the QC samples were 0.01, 0.25, 4, 10 and 100 μg/mL, prepared in the same way as the calibration sample. All of the stock solutions and the calibration and QC samples were immediately stored at −4 °C.

### 4.5. Sample Preparation

An aliquot of 50 μL of the plasma sample was placed in a 2 mL Eppendorf tube followed by the addition of 20 μL IS working solution (100 ng/mL), and was vortex-mixed for 30 s. The mixture was extracted with 1 mL ethyl acetate by vortex-mixing at a high speed for 10 min, and then the samples were centrifuged at 10,000 rpm for 10 min. The supernatant of 800 μL was transferred into new Eppendorf tubes and evaporated to dryness under vacuum at 50 °C. The dry residue was reconstituted with 200 µL of the mobile phase; after vortexing for 3 min, the mixture was transferred into 0.5 mL Eppendorf tubes and then centrifuged at 18,000 rpm for 10 min. A 5 µL aliquot of the supernatant obtained was then injected into the UPLC–MS/MS system for analysis. The preparation methods of the tissue homogenate, urine, feces and bile samples were the same as that of the plasma sample.

### 4.6. Validation of the Analytical Method

The methods for rat plasma, dog plasma, liver homogenate and urine were fully validated for specificity and selectivity, linearity, LLOQ, precision and accuracy, extraction recovery and the matrix effect; stability and dilution tests were performed according to the guidelines set by the United States Food and Drug Administration (FDA) for bioanalytical method validation. The analytical methods of the other tissues, feces and bile were partly validated (specificity, linearity, precision and accuracy, matrix effect, and dilution tests, etc.), because the homogenized rat tissue matrices had similar components and properties.

The selectivity was investigated by preparing and analyzing six samples of blank plasma (rat and dog) and other bio-samples at random. Each blank sample was tested using the above-described sample preparation procedure and UPLC–MS/MS chromatographic conditions in order to ensure no interference with HY-071085 and IS from the blank samples. The typical chromatograms of the blank plasma, medicated plasma, standard and plasma samples after administration from six rats are shown in Figure S5. The retention times of HY-071085 and IS were about 2.2 min and 2.0 min, respectively. The peak shapes of HY-071085 and IS were good, and the baselines were stable.

The calibration curve consisted of eight calibration samples covering the range of rat and dog plasma; the liver homogenate was 1 ng/mL to 1000 ng/mL, and the range of urine was 4 ng/mL to 4000 ng/mL. In order to evaluate the linearity of the method, calibration curves were prepared. The calibration curves were obtained by plotting the peak-area ratios of HY-071085 to the IS (*y*) versus the concentrations of HY-071085 (*x*), using a weighted least-squares linear regression (the weighting factor used was 1/*x*^2^). The lower limit of quantification (LLOQ) was defined as the lowest concentration on the calibration curves, which could be quantified reliably with acceptable accuracy (80–120%) and precision (<20%). The LLOQ was established using six samples that were independent of the standards. The standard curve data of HY-071085 in rat plasma are shown in Table S1. The other biological samples had good linearity in the range.

It was found that there were no residual effects in the retention times of HY-071085 or IS in the three blank samples. The recoveries and matrix effects of HY-071085 in rat plasma are summarized in Tables S2 and S3. After the sample preparation, the recoveries of HY-071085 in rat plasma were more than 90%. The matrix effects of HY-071085 were in the range of 95–107%, and the RSD of plasma from six different sources were less than 2.29%. The recoveries of HY-071085 in the other biological samples met the requirements, and there was no obvious matrix effect.

The intra- and inter-day precision and accuracy of LLOQ and the low-, medium- and high- concentration HY-071085 samples in rat plasma were investigated, as shown in Table S4. The precision (RSD) of the intra- and inter-day analysis of HY-071085 in rat plasma was within 4.97%, and the accuracy was 91.78–105.12%, including the LLOQ. The intra- and inter-day accuracy and precision of HY-071085 in other biological samples were within the required range. The results showed that the developed method had sufficient precision and accuracy for the quantification of HY-071085 in rat and dog plasma and other biological samples.

The stability of HY-071085 in rat plasma was evaluated by analyzing five repeated QC samples under various storage conditions: long-term freezing (freezing at −80 °C for 30 days), repeated freezing and thawing (thawing with a 37 °C water bath after freezing at −80 °C, repeated three times), normal temperature placement (a 37 °C constant temperature water bath for 8 h), and injector placement (4 °C for 24 h). The results showed that the HY-071085 in the rat blood samples was stable for the four treatment processes listed above, as shown in Table S5.

After the rat plasma containing 2 μg/mL HY-071085 was diluted five times, the measured accuracies were within the range of 95.25–105.25% of the nominal value, and the precision was 3.73%. The dilution effects of HY-071085 in other biological samples were within the required range. Therefore, the method could be used to quantify biological samples with HY-071085 concentrations exceeding the calibration range.

### 4.7. Pharmacokinetic Data Analysis

According to the obtained plasma concentration time data, the pharmacokinetic parameters were calculated using Phoenix WinNonlin 8.1 software. The pharmacokinetic parameters—i.e., the area under the curve (AUC), mean residence time (MRT), clearance rate (Cl/F) and steady-state apparent distribution volume (Vss/F)—were calculated by the method of moments, which was estimated according to the following formulae:

$$AUC_{tn}=\sum \left(C_{i}+C_{i-1}\right)\left(t_{i}-t_{i-1}\right)/2$$

$$AUC_{\infty }=AUC_{tn}+C_{m}/\lambda$$

$$AUMC_{tn}=\sum \left(t_{i}C_{i}+t_{i-1}C_{i-1}\right)\left(t_{i}-t_{i-1}\right)/2$$

$$AUMC_{\infty }=AUMC_{tn}+C_{tn}\left(t_{n}/\lambda +1/\lambda^{2}\right)$$

$$MRT=AUMC_{\infty }/AUC_{\infty }$$

Cl/F=Dose/AUC

$$V_{ss}/F=Cl*MRT$$

where *C_tn_* is the plasma concentration of *tn* at the last point after administration, and the elimination rate constant was obtained by ln(concentration) time linear regression.

Due to the drug having significant individual differences in rats, it could not be uniformly fitted with a PK atrioventricular model; as such, the pharmacokinetic parameters analyzed in this study were calculated using a non-atrioventricular model. The pharmacokinetic parameters are defined as follows: C_max_ is the maximum blood concentration after administration and absorption, T_max_ is the time required to reach C_max_, t_1/2_ is the time required to eliminate half of the plasma drug concentration after the drug distribution in the body reaches equilibrium, Cl/F is the apparent clearance rate, AUC_0-tn_ is the area under the blood concentration time curve within the dosing interval, AUC_0-∞_ is the area under the plasma concentration time curve from 0 to infinity, MRT is the average time that all drug molecules stay in the body, and Vss/F is the steady-state apparent distribution volume, which is the product of the clearance rate and average residence time.

## 5. Conclusions

Our studies investigated the pharmacokinetics of HY-071085 in healthy rats and beagle dogs after a single gavage, repeated gavages and intravenous injection. The concentration of HY-071085 in different biosamples was quantitatively detected using LC-MS/MS. The study results regarding the pharmacokinetic parameters, tissue distribution, excretion and plasma protein binding rate of HY-071085 were obtained. There were gender differences in the kinetic behavior of HY-071085 in rats; however, there were no differences in dogs. The calculated absolute oral bioavailability of HY-071085 in male and female rats was 2.8% and 10.8%, respectively, and was about 13.1% in beagle dogs. With regard to the tissue distribution, we found that HY-071085 was mainly distributed in most of the tissues except the brain, with the highest content in the gastric organs and intestine. In addition, less than 1.3% of the HY-071085 was excreted in feces, urine and bile, indicating that nearly all of the HY-071085 was either absorbed or excreted in the form of its metabolites. The results of this study will provide a more complete understanding of the absorption, distribution, and excretion of limonin analogues.

## Figures and Tables

**Table 1:** Pharmacokinetic parameters of HY-071085 after single-dose intravenous and intragastric administration, and repeated-dose intragastric administration in rats (mean ± SD).

Parameters	Rat											
	S-IG (3.25 mg/kg)		S-IG (6.5 mg/kg)		S-IG (13 mg/kg)		S-IV (3.25 mg/kg)		R-IG (6.5 mg/kg)			
	Male	Female	Male	Female	Male	Female	Male	Female	Male 14 Days	Female 14 Days	Male 28 Days	Female 28 Days
C_max_ (ng/mL)	11.33 ± 4.54	20.42 ± 9.08	107.36 ± 139.23	145.32 ± 64.54	380.80 ± 152.47	761.40 ± 321.71 *	-	-	63.20 ± 11.17	124.94 ± 80.28	38.86 ± 12.71	37.24 ± 19.93 ^#^
T_max_ (h)	0.22 ± 0.04	0.70 ± 0.73	0.45 ± 0.59	0.90 ± 0.68	0.85 ± 0.45	0.95 ± 0.51	-	-	0.65 ± 0.38	0.68 ± 0.75	0.60 ± 0.78	0.33 ± 0.16
AUC_0-tn_ (h·ng/mL)	20.82 ± 3.01	73.74 ± 25.31	107.54 ± 54.17	420.99 ± 205.42 *	854.77 ± 290.22	2079.61± 407.39 **	738.95 ± 146.40	682.45 ± 224.58	126.81 ± 22.45	228.83 ± 159.42	55.00 ± 8.82	65.83 ± 28.92 ^#^
AUC_0-∞_ (h·ng/mL)	-	-	125.13 ± 72.28	428.74 ± 204.08 *	-	-	740.28 ± 146.42	683.92 ± 39.25	132.59 ± 22.26	232.87 ± 158.92	59.85 ± 10.53	70.15 ± 28.76 ^#^
Cl/F (L/h/kg)	-	-	70.66 ± 43.65	18.13 ± 8.07 *	-	-	4.50 ± 0.71	4.76 ± 0.27	50.13 ± 8.32	37.82 ± 20.40	111.46 ± 20.58	103.69 ± 35.13 ^#^
MRT (h)	3.48 ± 1.98	4.06 ± 0.82	2.89 ± 1.87	2.57 ± 0.64	1.94 ± 0.39	2.29 ± 0.92	0.29 ± 0.04	0.34 ± 0.05	2.26 ± 0.33	2.18 ± 0.27	1.74 ± 0.86	2.54 ± 1.15
Vss/F (L/kg)	-	-	172.12 ± 70.35	36.31 ± 23.24 **	-	-	1.31 ± 0.18	1.63 ± 0.17 *	108.46 ± 13.81	79.97 ± 51.00	168.72 ± 35.19	291.76 ± 251.13
t_1/2_ (h)	2.35 ± 1.27	2.16 ± 1.13	1.98 ± 1.04	1.30 ± 0.36	1.21 ± 0.31	0.90 ± 0.42	0.40 ± 0.10	0.34 ± 0.05	1.52 ± 0.23	1.38 ± 0.24	1.11 ± 0.41	1.75 ± 1.14

* *p* < 0.05, ** *p* < 0.01 compared with male rats (*t*-test); ^#^ *p* < 0.01 compared with a single dose (*t*-test); S-IV: single-dose intravenous; S-IG: single-dose intragastric; R-IG: repeated-dose intragastric.

**Table 2:** Pharmacokinetic parameters of HY-071085 after single-dose intravenous and intragastric administration, and repeated-dose intragastric administration in beagle dogs (mean ± SD).

Parameters	Dog				
	S-IG (mg/kg)			S-IV (mg/kg)	R-IG (mg/kg)
	1.25	1.25	5	1.25	2.5
C_max_ (ng/mL)	37.9 ± 20.05	1202.70 ± 1506.40	435.68 ± 205.91	1202.70 ± 1506.40	445.00 ± 135.32 ^#^
T_max_ (h)	0.49 ± 0.43	0.09 ± 0.03	0.69 ± 0.55	0.09 ± 0.03	0.27 ± 0.15
AUC_0-tn_ (h·ng/mL)	51.13 ± 24.92	591.16 ± 538.62	606.04 ± 236.49	591.16 ± 538.62	723.23 ± 269.96 ^#^
AUC_0-∞_ (h·ng/mL)	54.06 ± 25.37	593.60 ± 538.37	609.98 ± 235.31	593.60 ± 538.37	728.57 ± 270.64 ^#^
Cl/F (L/h/kg)	31.00 ± 20.44	3.07 ± 1.45	9.38 ± 3.66	3.07 ± 1.45	1.94 ± 0.72 ^#^
MRT (h)	1.34 ± 0.53	0.62 ± 0.20	1.46 ± 0.49	0.62 ± 0.20	1.58 ± 0.14 ^#^
Vss/F (L/kg)	35.26 ± 19.11	2.07 ± 1.17	11.52 ± 6.03	2.07 ± 1.17	3.04 ± 1.19 ^#^
t_1/2_ (h)	0.88 ± 0.44	0.70 ± 0.09	0.83 ± 0.13	0.70 ± 0.09	1.08 ± 0.13 ^#^

# *p* < 0.01 compared with a single dose (*t*-test); S-IV: single-dose intravenous; S-IG: single-dose intragastric; R-IG: repeated-dose intragastric.

**Table 3:** Material balance of the rats after the gavage of 6.5 mg/kg HY-071085 (mean ± SD).

Tissue	HY-071085 Prototype
Feces	0.812 ± 0.611%
Urine	0.254 ± 0.164%
Bile	0.206 ± 0.206%
Total	1.27%

**Table 4:** Comparison of the plasma protein binding rate of HY-071085 in rats, dogs and humans.

	Concentration (ng/mL)	50	100	200	400
Dog	Mean%	37.81	32.86	42.18	45.46
	SD%	2.60	7.84	4.60	4.38
Human	Mean%	100.00	73.28	67.48	61.56
	SD%	0.00	6.39	1.48	3.17
Rat	Mean%	73.93	75.25	77.17	78.86
	SD%	1.35	1.83	2.77	0.41

**Table 5:** The pharmacokinetics study design using rats.

Serial number	Sex	Age	Drug	Route	*n*	Dose (mg/kg)	Sampling Time
1~5	M	6 weeks	HY-071085	S-IG	5	3.25	Blood: 5, 10, 15, 30, 45, 60, 90, 120, 360, 480 min
6~10	F	6 weeks	HY-071085	S-IG	5	3.25	
11~15	M	6 weeks	HY-071085	S-IG	5	6.5	
16~20	F	6 weeks	HY-071085	S-IG	5	6.5	
21~25	M	6 weeks	HY-071085	S-IG	5	13	
26~30	F	6 weeks	HY-071085	S-IG	5	13	
31~35	M	6 weeks	HY-071085	S-IV	5	3.25	Blood: 2, 5, 15, 30, 45, 60, 90, 120, 180, 240, 360 min
36~40	F	6 weeks	HY-071085	S-IV	5	3.25	
41~45	M	6 weeks	HY-071085	R-IG	5	6.5	Blood 14 days and 28 days: 0.083, 0.167, 0.25, 0.75, 1, 1.5, 2, 4, 6, 8 h
46~50	F	6 weeks	HY-071085	R-IG	5	6.5	
51~55, 61~65, 71~75	M	6 weeks	HY-071085	S-IG	15	13	Tissue: 10 min, 45 min, 4 h
56~60, 66~70, 76~80	F	6 weeks	HY-071085	S-IG	15	13	
81~85	M	6 weeks	HY-071085	S-IG	5	6.5	Urine, Feces: 0~6 h, 6~12 h, 12~24 h
86~90	F	6 weeks	HY-071085	S-IG	5	6.5	
91~95	M	6 weeks	HY-071085	S-IG	5	6.5	Bile: 0~2 h, 2~4 h, 4~6 h, 6~8 h
96~100	F	6 weeks	HY-071085	S-IG	5	6.5	

**Table 6:** The pharmacokinetics study design using beagle dogs.

Group		Sex	Age	Drug	Single-Dose Crossover Cycle				R-IG Dose (mg/kg)	Blood Sampling Time
					I Dose (mg/kg)	II Dose (mg/kg)	III Dose (mg/kg)	IV Dose (mg/kg)		
A	1	F	7–9 months	HY-071085	S-IG (1.25)	S-IG (5)	S-IG (2.5)	S-IV (1.25)	2.5	S-IG: 5, 10, 15, 30, 45, 60, 90, 120, 240, 360, 480 min S-IV: 2, 5, 15, 30, 45, 60, 90, 120, 240, 360, 480 min R-IG 7 d: 5, 10, 15, 30, 45, 60, 90, 120, 240, 360, 480 min
	2	M							2.5	
B	3	F	7–9 months	HY-071085	S-IG (2.5)	S-IV (1.25)	S-IG (5)	S-IG (1.25)	2.5	
	4	M							2.5	
C	5	F	7–9 months	HY-071085	S-IG (5)	S-IG (1.25)	S-IV (1.25)	S-IG (2.5)	2.5	
	6	M							2.5	
D	7	F	7–9 months	HY-071085	S-IV (1.25)	S-IG (2.5)	S-IG (1.25)	S-IG (5)	2.5	
	8	M							2.5	

## Data Availability

Data is contained within the article and supplementary material.

## Supplementary Materials

The following supporting information can be downloaded at: https://www.mdpi.com/article/10.3390/ph15070801/s1. Figure S1: Cmax–dose curve of HY-071085 after the single-dose intragastric administration of 3.25, 6.5 and 13 mg/kg HY-071085 in rats (A: constant coordinates; B: double logarithmic coordinates). Figure S2: AUC0-∞–dose curve of HY-071085 after the single-dose intragastric administration of 3.25, 6.5 and 13 mg/kg HY-071085 in rats (A: constant coordinates; B: double logarithmic coordinates); Figure S3: AUC0-∞–dose curve of HY-071085 after the single-dose intragastric administration of 1.25, 2.5 and 5 mg/kg HY-071085 in beagle dogs (A: constant coordinates; B: double logarithmic coordinates). Figure S4: Cmax–dose curve of HY-071085 after the single-dose intragastric administration of 1.25, 2.5 and 5 mg/kg HY-071085 in beagle dogs (A: constant coordinates; B: double logarithmic coordinates). Figure S5: Specificity chromatogram of HY-071085 in rat plasma (A: rat blank plasma; B: 50 ng/mL HY-071085 and internal standard theophylline standard solution; C: after adding HY-071085 into rat blank plasma, the concentration in plasma was 1 ng/mL and internal standard theophylline; D: plasma samples of rats after the intragastric administration of 6.5 mg/kg HY-071085 for 5 min). Table S1: Standard curve data of HY-071085 in rat plasma (*n* = 3). Table S2: Recovery of HY-071085 in rat plasma. Table S3: Matrix effects of HY-071085 and the internal standard theophylline in rat plasma. Table S4: HY-071085 intra- and inter-batch accuracy and precision. Table S5: Test results of the stability of HY-071085 in plasma after long–term freezing, repeated freeze–thawing, normal temperature (8 h), and injector (24 h) (*n* = 5). Table S6: Comparison of the physical and chemical properties of limonin and HY-071085.
