# Supplementary material for: Preclinical Drug Pharmacokinetic, Tissue Distribution and Excretion Profiles of the Novel Limonin Derivate HY-071085 as an Anti-Inflammatory and Analgesic Candidate in Rats and Beagle Dogs
Source: Pharmaceuticals (Basel). 2022 Jun 27;15(7):801. doi: 10.3390/ph15070801 (PMC9316000; doi:10.3390/ph15070801)
Supplement: Supplementary file 1 [file pharmaceuticals-15-00801-s001.zip › pharmaceuticals-1773045-supplementary.pdf]

Supplementary Materials

Table S1. Standard curve data of HY-071085 in rat plasma (n=3).

Concentration（ng/mL）	As/Ai				
	L1	L2	L3	Mean	SD
1	0.013	0.012	0.013	0.013	0.001
2	0.024	0.023	0.024	0.024	0.001
5	0.057	0.057	0.058	0.057	0.001
20	0.23	0.23	0.23	0.23	0.00
50	0.57	0.56	0.56	0.56	0.006
200	2.30	2.37	2.30	2.32	0.04
500	5.51	5.59	5.45	5.52	0.07
1000	10.7	11.0	10.7	10.80	0.17
a	0.0112	0.0113	0.0111	0.0112	0.0001
b	0.00169	0.000574	0.00173	0.0013	0.0007
r	0.9997	0.9997	0.9997	-	-

Table S2. Recovery of HY-071085 in rat plasma.

Concentration（ng/mL）	As/Ai	Rf	SD	As/Ai	Rc	SD	Recovery (%)
	0.022			0.028			
	0.023			0.026			
2	0.023	0.023	0.00039	0.024	0.025	0.0016	90.81
	0.023			0.024			
	0.024			0.024			
	0.54			0.59			
	0.53			0.59			
50	0.55	0.54	0.0079	0.56	0.59	0.021	91.05
	0.54			0.62			
	0.54			0.61			
	8.18			8.80			
	8.57			8.85			
800	8.35	8.46	0.18	8.76	8.89	0.17	95.16
	8.70			9.22			
	8.50			8.82			

Table S3. Matrix effects of HY-071085 and internal standard theophylline in rat plasma.

Concentration（ng/mL）	ARi	As/Ai	Ac	ARi/ Ac	Mean	SD	RSD%
	0.026	0.036		0.98			
	0.026	0.025		0.97			
2	0.026	0.025	0.027	0.95	0.97	0.013	1.34
	0.026	0.025		0.97			
	0.027	0.025		0.98			
	0.026	0.025		0.95			
800	8.63	8.68	8.49	1.02	1.04	0.024	2.29
	9.08	8.38		1.07			

8.57	8.55	1.01
8.95	8.51	1.05
9.02	8.33	1.06
8.99	8.56	1.06

Table S4. HY-071085 intra- and inter-batch accuracy and precision.

Batch	Concentration (ng/mL)	Intra-day (n=5)				Inter-day (n=15)			
		Mean	SD	RSD (%)	Accuracy (%)	Mean	SD	RSD (%)	Accuracy (%)
1	1	0.97	0.03	3.02	97.18	0.95	0.03	3.50	94.57
	2	1.91	0.04	1.89	95.30	1.91	0.07	3.42	95.73
	50	48.34	0.73	1.50	96.68	50.51	1.40	2.74	101.01
	800	760.4	16.16	2.12	95.05	764.47	13.33	1.74	95.56
2	1	0.92	0.02	2.50	91.78	-	-	-	-
	2	2.00	0.09	4.43	100.10	-	-	-	-
	50	50.62	1.89	3.73	101.24	-	-	-	-
	800	757.2	6.85	0.91	94.65	-	-	-	-
3	1	0.95	0.05	4.97	94.74	-	-	-	-
	2	1.84	0.07	3.95	91.80	-	-	-	-
	50	52.56	1.58	3.00	105.12	-	-	-	-
	800	775.8	16.98	2.19	96.98	-	-	-	-

Table S5. Test results of stability of HY-071085 in plasma at long-term freezing, repeated freeze-thaw, normal temperature (8h) and injector (24h) (n=5).

Concentration (ng/mL)	Long-term freezing		Repeated freeze-thaw		Normal temperature (8h)		Injector (24h)	
	2	800	2	800	2	800	2	800
Mean	2.25	812.00	1.95	778.60	2.12	781.60	2.10	812.40
SD	0.10	26.56	0.03	14.07	0.08	18.71	0.08	15.00
RSD (%)	4.25	3.27	1.57	1.81	3.84	2.39	4.02	1.85
Accuracy (%)	112.40	101.50	97.40	97.33	105.90	97.70	105.00	101.55

Table S6. Comparison of physical and chemical properties between limonin and HY-071085.

Physicochemical properties	Limonin	HY-071085
Melting point	298℃	138-141℃
Intrinsic solubility (Temp 25℃)	0.22 mg/mL	4.5 mg/mL
pKa	-	6.11
LogD (Temp 25℃)	(pH 7.0) 0.47	(pH 7.4) 2.45
logP (Temp 25℃)	0.474	2.8023

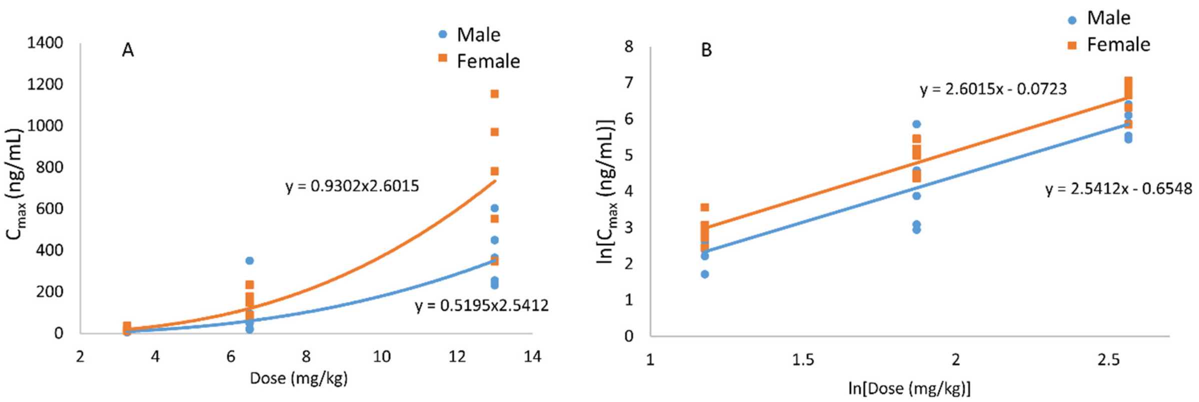

Figure S1. C_{max}-dose curve of HY-071085 after single-dose intragastric of 3.25, 6.5 and 13 mg/kg HY-071085 in rats. (A: constant coordinates; B: double logarithmic coordinates)

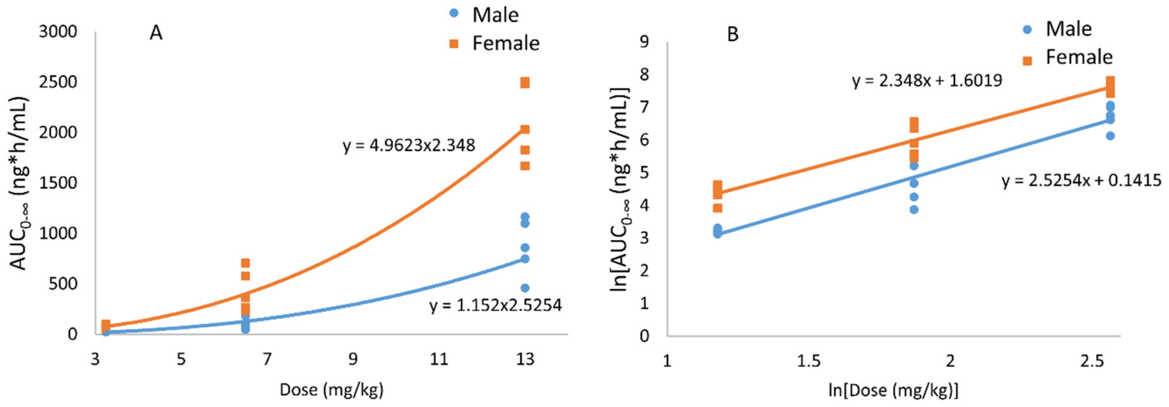

Figure S2. $AUC_{0-\infty}$-dose curve of HY-071085 after single-dose intragastric of 3.25, 6.5 and 13 mg/kg HY-071085 in rats. (A: constant coordinates; B: double logarithmic coordinates)

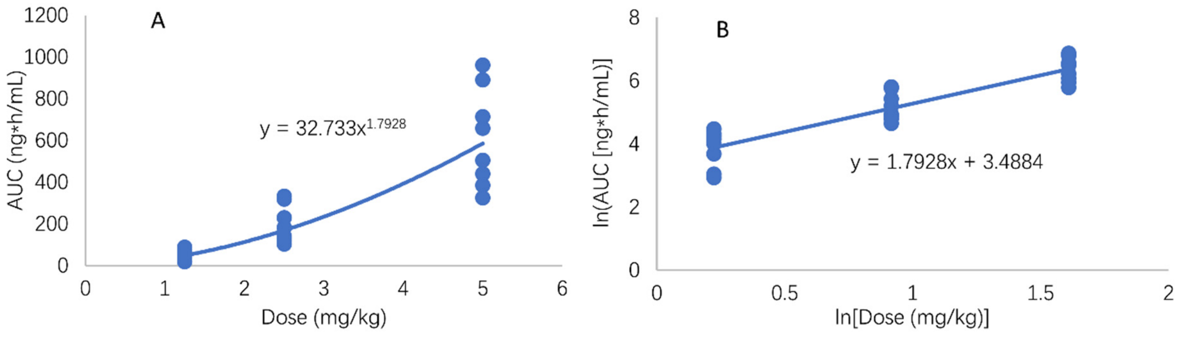

Figure S3. $AUC_{0-\infty}$-dose curve of HY-071085 after single-dose intragastric of 1.25, 2.5 and 5 mg/kg HY-071085 in beagle dogs. (A: constant coordinates; B: double logarithmic coordinates)

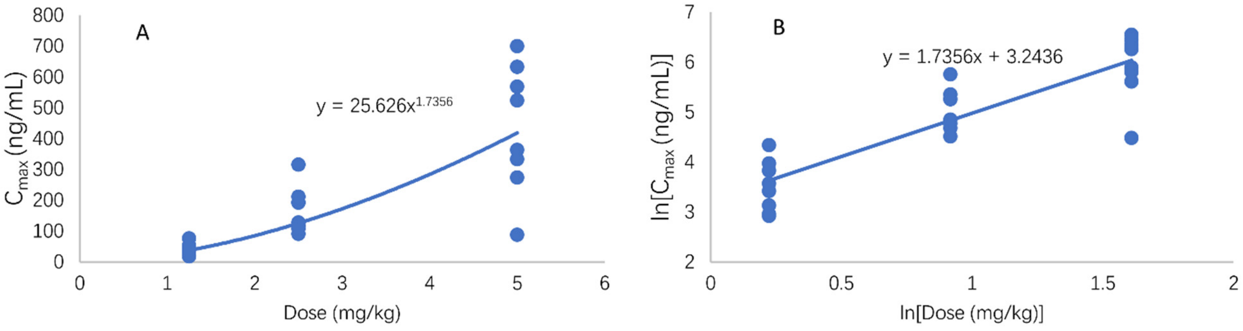

Figure S4. C_{max}-dose curve of HY-071085 after single-dose intragastric of 1.25, 2.5 and 5 mg/kg HY-071085 in beagle dogs. (A: constant coordinates; B: double logarithmic coordinates)

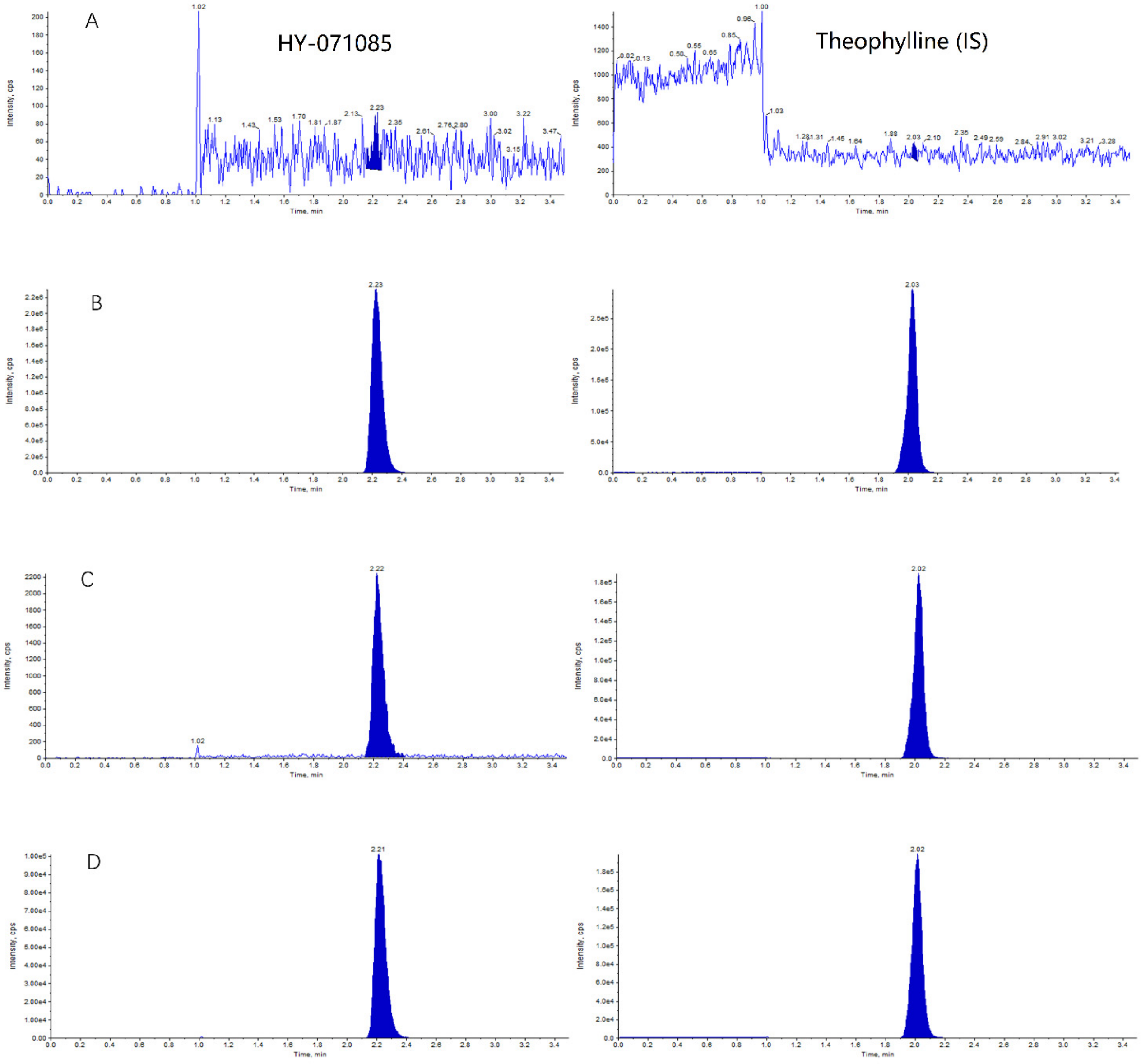

Figure S5. Specificity Chromatogram of HY-071085 in rat plasma.

(A. Rat blank plasma; B. 50 ng/mL HY-071085 and internal standard theophylline standard solution; C. After adding HY-071085 into rat blank plasma, the concentration in plasma was 1 ng/mL and internal standard theophylline; D. Plasma samples of rats after intragastric administration of 6.5 mg/kg HY-071085 for 5 min）
